# The unfolded von Willebrand factor response in bloodstream: the self-association perspective

**DOI:** 10.1186/1756-8722-5-65

**Published:** 2012-10-15

**Authors:** Hailong Yuan, Ning Deng, Songmei Zhang, Yange Cao, Qiong Wang, Xin Liu, Qing Zhang

**Affiliations:** 1 Key Laboratory of Biocontrol, School of Life Sciences, Sun Yat-sen University, Guangzhou, P R China; 2 Key Laboratory of Molecular Immunology and Antibody Engineering of Guangdong Province, Antibody Engineering Center in Jinan University, Guangzhou, P R China; 3 Sun Yat-sen Institute of Hematology, Sun Yat-sen University, Guangzhou, P R China

**Keywords:** von Willebrand factor, Self-association, Shear force, Thiol/disulfide exchange, Signaling molecules

## Abstract

von Willebrand factor (vWF) is a multimeric glycoprotein essential for hemostasis after vascular injury, which modulates platelet-surface and platelet–platelet interactions by linking platelet receptors to the extracellular matrix and to each other. The crucial role of vWF in platelet function is particularly apparent when hemodynamic conditions create blood flow with high shear stress. Through multiple functional domains, vWF mediates the attachment of platelets to exposed tissues, where immobilized vWF is able to support a homotypic and/or heterotypic self-association. The self-association of vWF is also supported by a rapidly expanding reservoir of novel evidences that the thiol/disulfide exchange regulates vWF multimer size in the blood circulation. Moreover, in addition to proteolysis and reduction of ADAMTS13 (a disintegrin and metalloproteinase with a thrombospondin type 1 motif, member 13), the regulation of vWF multimer size and self-association may depend on a disulfide bond reductase activity ascribed to thrombospondin-1 (TSP-1). Along with the classical signaling pathways in activated platelets, evidence is emerging that lipid rafts also play important roles in various phases of hemostasis and thrombosis and facilitate the interaction between the key signaling molecules. Developments in these areas will refine our understanding of the role played by vWF self-association in physiological hemostasis and pathological thrombosis.

## Introduction

vWF is a large multimeric plasma glycoprotein that plays an important role in hemostasis and thrombosis. This protein is encoded by a very large gene (180 kb, 52 exons) located at the tip of the short arm of chromosome 12, region 12p12-l2pter [1,2]. vWF is synthesized by megakaryocytes and endothelial cells as pre-pro-vWF. The pre-pro-vWF, composed of a 22-amino acid signal peptide, a 741-amino acid propeptide and the 2050-amino acid mature protein [3], undergoes extensive post-translational processing, glycosylation and assembly in the endoplasmic reticulum, Golgi and post-Golgi [3-5]. vWF multimers synthesized in endothelial cells or in megakaryocytes are stored in cytoplasmic granules, which are also called the Weibel-Palade bodies in endothelial cells [6,7] or at the periphery of platelet α-granules [8]. The mature monomeric protein is a ~250 kDa molecule containing 12 N-linked and 10 O-linked oligosaccharide chains [9]. Analysis of the amino acid sequence shows that each vWF monomer contains modular domains that are arranged in the order D’-D3-A1-A2-A3-D4-B1-B2-B3-C1-C2-CK. Each vWF monomer has binding sites for fibrin, platelet glycoprotein Ib, IIb/IIIa (GPIb, GPIIb/IIIa) and clotting factor VIII (FVIII) [3]. In the flow of circulation, large bundles or filaments of disulfide-linked vWF multimers of 500 to 20,000 kDa have been found [10]. It is believed that under normal conditions the size of vWF multimers is precisely controlled by ADAMTS13-mediated proteolysis of the Tyr1605-Met1606 peptide bond in the A2 domain [11,12]. Genetic or acquired deficiency of ADAMTS13 causes thrombotic thrombocytopenic purpura (TTP), a life-threatening disease, in which microvascular thrombi forms in arterioles and capillaries [10], while a paucity of large vWF multimers caused by mutations in the A2 domain is associated with the bleeding disorder, type 2A von Willebrand disease (vWD) [3,13,14]. Previously, there were a number of studies that focused on the role of ADAMTS13 in regulating vWF size under hydrodynamic shear [15]. Recently, increasing amount of evidences suggest the self-association of vWF may be an additional mechanism regulating the protein size in circulation, implying a critical impact of vWF self-association on hemostasis and thrombosis [16-18]. However, little is known about the mechanism by which plasma vWF self-associates under high shear stress at site of vascular rupture. This article reviewed recent literature on vWF self-association and we provide a summary model to explain dynamic regulation of vWF self-association (by multidomain interaction, hydrodynamic shear force or thiol/disulfide exchange) and its effect on the complex signal pathways of platelet activation.

### Functional domain of vWF and the interaction with platelet

The vWF subunit comprises several domains of which specific functions have been identified [3]. The D’-D3 domains are potential binding sites for P-selectin [19,20] and are involved in vWF multimerization [21]. Much of the functional activity of vWF multimers resides within the three tandem A domains, A1, A2, and A3. The binding of the A1 domain to GPIb, which initiates rolling interactions, is the first step in the formation of a hemostatic plug at the site of vascular injury [22]. Moreover, the A1 domain has binding sites for heparin, the bacterial glycopeptide antibiotic ristocetin, the snake venom botrocetin and collagen [3,23-28]. The A2 domain situated between A1 and A3 domain lacks long-range disulphide bonds. The cleavage of force-sensing A2 domain by ADAMTS13 depends on hydrodynamic shear force-dependent unfolding of the A2 domain, and occurs at the Tyr1605–Met1606 bond within the A2 domain [11]. The size of the vWF multimers is modulated by the ADAMTS13 domain, depending on the rheological forces and calcium ion [29]. The A3 domain is the binding site for fibrillar collagen type I and III [25,26,30]. The structural change of A3 domain enables the binding of the A1 domain to GPIb consisting of a disulfide-linked α chain and β chain [31,32]. The initial step mediated by A1 domain is followed with platelet activation and stable adhesion via the irreversible interaction between C domain and GPIIb/IIIa by RGDS within the C domain, which initiates and expands the platelet aggregation cascade [22,33,34]. The vWF C1 and C2 domains are involved in platelet adhesion to polymerized fibrin at high shear stress [35]. The CK domain is a prerequisite to vWF tail-to-tail dimerization [36]. Thus, the functional domains within vWF are potential elements that mediate intradomain or interdomain interaction contributing to vWF self -association.

### Multiple domains interaction contributing to vWF self-association

**Figure 1:**
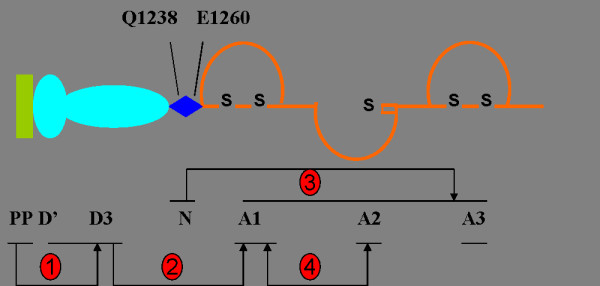
**Potential intradomain interaction of vWF.** Partial structure of pro-vWF is shown from vWFpp to vWF A3 domain. ①vWFpp binding to the D’D3 domain of mature vWF occurs in the circulation and attenuates platelet adhesion and activation. ②The D’D3 domain of von Willebrand factor shields the A1 domain in its resting conformation and negatively regulates the binding of A1 domain to GPIb. ③N-terminal flanking region of A1 domain (amino acids Gln1238-Glu1260) in von Willebrand factor stabilizes the structure of A1A2A3 complex and modulates platelet activation under shear stress. ④The recombinant A2 domain of von Willebrand factor specifically binds to the active conformation of A1 domain and blocks the interaction with GPIb.

vWF self-association was first reported by Savage *et al*. [37] who showed that fluid-phase vWF multimers could homotypically associate with vWF multimers that were immobilized onto a collagen surface or glass. These self-associated vWF multimers supported platelet adhesion under shear stress and vWF multidomain interaction potentially contributes to vWF self-association under shear force. Numerous studies have been carried out in order to identify the exact domains that contribute to the homotypical self-association. It has been shown that vWF self-association may involve the vWF propeptide (vWFpp), D’D3, N-terminal flanking region of A1 domain, A1 and A2 domain (Figure 1). However, the potential function of the A3 domain in homotypical association is questioned, as blocking or devoiding A3 domain in both soluble and immobilized wild type vWF (WT-vWF) contributed to the same platelet adhesion as that observed when soluble and immobilized WT-vWF were used in a flow studies [37]. A close relationship between the A1 and A2 domains in vWF has also been demonstrated. For instance, one study suggested that the A1 domain inhibited the cleavage of the A2 domain, and that the interaction of platelet GPIbα or heparin with the A1 domain terminated such inhibition, thus making the A1A2A3 domain more susceptible to cleavage by ADAMTS13 [38]. Another study has advanced our understanding of intradomain interaction by showing that the recombinant A2 domain polypeptide can specifically recognize the GPIbα-binding conformation in the A1 domain activated by the modulator ristocetin or immobilized vWF, indicating that intradomain interactions impeded the accessibility of the A1 domain. Interestingly, in contrast to plasma vWF, the A2 domain bound to the ultra-large vWF (ULvWF) multimers or a recombinant vWF-A1A2A3 polypeptide containing a gain-of-function mutation (R1308 L) of type 2B vWD in the absence of ristocetin [39]. 

It has been recently shown that the only binding site for vWFpp in mature vWF is in its D’D3 domain and vWFpp has the ability of partially reducing platelet adhesion and activation by reduce the binding affinity of the VWF-A1 domain for platelet GpIbα [40,41]. The results presented in this study demonstrated that deletion of the D’D3 region in vWF facilitated the binding of vWF to GPIbα, suggesting an inhibitory role for this region. The isolated D’D3 region inhibited the GPIbα interaction with a vWF deletion mutant lacking this region, indicating that intradomain interactions limit the accessibility of the A1 domain. Interestingly, N-terminal region of the A1 domain (amino acids Gln1238-Glu1260) was also reported to modulates the interaction between vWF and GPIbα [42]. The N-terminal region of vWF between the D4 and cystine-knot domains was visualized for the first time to form a rod at the acidic pH of the trans-Golgi and Weibel-Palade bodies. In addition, the A2, A3 and D4 domains were observed to assembly a raceme with three pairs of opposed, large, flower-like domains by electron microscopy [43]. These observations shed light on the interdomain interaction attributing to vWF self-association that occurs during the biogenesis of vWF prior to its secretion into the vasculature. In addition to mimicking the in vivo environments for vWF assembly, there were reports that the proteolytic vWF-fragments SpII (1366–2050) and SpIII (1–1365) respectively inhibited the interaction between vWF and biotinylated vWF (b-vWF) by 70% and 80%, as measured by ELISA analysis, suggesting that SpII and SpIII contributed to vWF self-association [44]. An interesting hypothesis was proposed that the same multiple-domain interaction accounted for the stabilization of the globular structure of vWF under rest state. Once activated by immobilizing or shear force, homotypic and/or heterotypic region self-association might occur [44]. Despite extensive studies, however, the mechanism by which intradomain interaction mediates vWF self-association with each other has not been clearly elucidated. Further studies are thus required in order to identify the precise nature of the intradomain or interdomain interaction attributing to vWF self-association.

### Shear force and functional self-association of vWF

**Figure 2:**
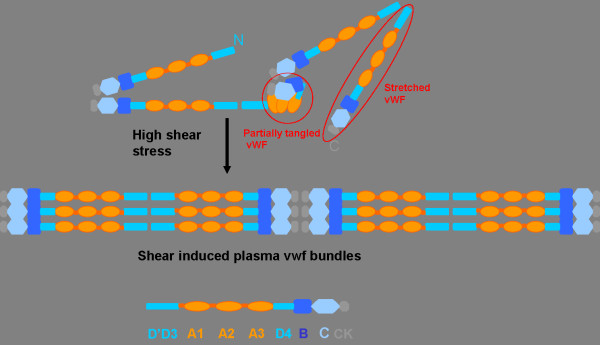
**Model for regulation of plasma vWF multimers self-association under shear stress.** Plasma vWF multimers circulated in blood are folded into tangled and condensed coils. In this folded conformation, the intradomain interaction may block the A domain and prevent the bindings of vWF to platelet receptors. Under the high shear stress, globular plasma vWF multimers are stretched to covalently associate with each other. The lateral associations can seek to maximize their exposure in order to augment binding capacity and bond strength for platelet GPIb in multiple vWF A1 domains.

Hemostasis and thrombosis have significance beyond the scope of blood diseases, and they have been extensively studied in the context of the dynamic nature of the protein and hemorheology. The shear force in blood circulation is the crucial determinant of vWF self-association in different systems. vWF undergos a self-association process when the conformational state of vWF is changed by chemical potential of allosteric effector such as ristocetin, even in the absence of hydrodynamic shear stress and protein adsorbing surface [44,45]. The mechanism of ristocetin induced self-association might be related to the ability of ristocetin to specifically bind to the A1 domain of vWF and trigger a large conformational change of the vWF molecule, which is responsible for its binding to platelet GpIbα [46]. On the other hand, individual molecules can self-associate not only under shear force [37,47,48], but also in a static condition [49]. It was shown previously that soluble VWF multimers isolated from human plasma self-assemble to a network of fibers immobilized on a collagen matrix as observed under immunofluorescence microscopy and were functionally active to bind platelets under the condition of the shear flow [47]. Atomic force microscopy images were then used to elucidate the nanostructure of VWF fibers and illustrate self-association and -aggregation of several filamentous multimers [47]. Similarly, the nature of the assembly that is formed by VWF self-association in solution induced by ristocetin binding was clarified by atomic force microscopy [45]. vWF was shown to adhere to the collagen coated surface at high shear rates also form a spider web like network, which represents a very adhesive substrate for blood platelets [50]. This team monitored the adsorption process of multiple vWF fibers on an adsorbing collagen substrate in the planar micro-fluid device. They were able to follow the formation of an immobilized network that constitutes a “sticky” grid necessary for blood platelet adhesion under high shear flow. At the same time, Shankaran *et al*. have made a significant contribution to our understanding of the process of vWF self-association in suspension induced by the hydrodynamic forces. This study applied western blotting and densitometry analysis to confirm the occurrence of larger vWF moieties in sheared samples, suggesting that vWF self-associated under hydrodynamic shear [51]. Also, flow studies revealed that soluble vWF over a vWF-coated surface underwent dynamic and reversible “homotypic” self-association that facilitated platelet adhesion, whereas immobilized BSA, human fibrinogen, and fibronectin could not substitute for vWF in this process [52]. Recent experimental polymer physics studies had paid significant attention to the mechanism by which vWF unfolded to self-associate at the sites of vascular injury [53-55]. vWF stretching was further elaborated from a polymer physics perspective to demonstrate that elongation flows, which appear during vasoconstriction or stenosis, were the most principal factors in the regulation of vWF, and consequently one of the prominent triggers in blood clotting [56]. In vivo, shear force could promote conformational changes in vWF and enable vWF to interact with subendothelial proteins and platelets, thus promoting primary hemostasis. Shear force might act as a key factor in the dynamic regulation of vWF self-association. This, together with the in vitro and in vivo data, makes a compelling case for shear force having a biologically relevant role in the control of vWF self-association (Figure 2) 

Base on the above findings and considerations, some studies validated that purified plasma vWF multimers significantly increased the numbers of strings on histamine-stimulated human umbilical vein endothelial cells (HUVECs) under flow conditions [57]. It is noteworthy that the sheared plasma vWF molecules resemble polydispersed coils in contrast to the rodlike morphology of unsheared samples [51]. Recently, vWF was observed to transit from a loosely coiled ball to a stretched structure in response to shear force. The unfolding process was reversible since once the hydrodynamic shear force was reduced to 5,000s^-1^, vWF immediately relaxed back to its compact state [58,59]. There is other evidence suggesting that relaxation process of stretched vWF bundles formed by hydrodynamic stress proceeded through hopping events between a multitude of minima. The longest relaxation time was found to be dominated by the internal conformations and effective friction within the bundle, consistent with current ideas of vWF self-association [60]. Up to now, little is known about the dynamic and mechanical characteristics of such vWF fibers. Physiological and pathological levels of shear stresses (50 and 100 dynes/cm^-2^) were demonstrated to modify the vWF adhesion activity by promoting the formation of disulfide bonds which may increase vWF multimer size [61]. It was proposed that high shear stress elongates globular plasma vWF multimers, promotes covalently association of vWF by forming interchain disulfide bonds, and increases binding avidity and bond strength for platelet GPIb.

### Thiol/disulfide exchange and vWF self-association

**Figure 3:**
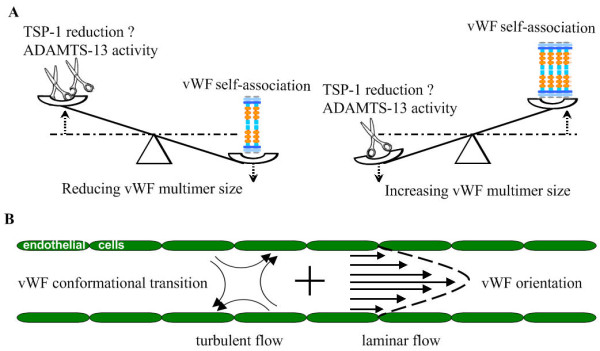
**Model for regulation of the balance of vWF self-association and two forms of flows in blood vessel.A**: The regulation of vWF multimer size equilibrates between reducing vWF multimer size and increasing vWF multimer size. **Reducing vWF multimer size:** In turbulent flow, the elongated vWF multimers are reduced by TSP-1 and ADAMTS13 due to the elevated activities of TSP-1 and ADAMTS13 (cleavage and reduction activity) in response to turbulent flow and can’t self-associate. **Increasing vWF multimer size:** In laminar flow, the elongated vWF multimers that tend to flow in parallel are undesirable substrates of TSP-1 and ADAMTS13, because some corresponding sites of vWF are buried in vWF fibers. It is speculated that the paralleled vWF multimers are more prone to self-associate to form vWF fibers. **B**: The turbulence and laminar flow are two of the complex and diverse forms of flow in blood. Turbulent flow is always highly irregular, in which the blood proteins tend to move in different directions at different speeds. vWF multimers undergo conformational change from a loosely coiled ball to an elongated structure in turbulent flow. Laminar flow in a cylindrical vessel can be visualized as a series of fluid layers with different velocity. The laminae close to the vessel wall have less velocity than those near the center (depicted schematically by arrows of different length). The elongated vWF multimers tend to be oriented to each other in laminar flow.

The regulation of vWF multimer size involves a series of coordinated and linked transitions, especially conformational changes of vWF regulated by blood flow and thiol/disulfide exchange. On the one hand, vWF multimers, which conformationally change from a loosely coiled ball to an elongated structure in response to turbulent flow, are the preferred substrate for TSP-1 and ADAMTS13. TSP-1 may reduce vWF multimers size by reducing the disulfide bonds. On the other hand, vWF multimers secreted by HUVECs can self-associate in diverse patterns, forming twisted bundles and networks on endothelial cells when exposed to laminar flow. The shear-mediated vWF reassembling into larger structures may represent an efficient way to allow locally the presence of molecular species needed for thrombus formation (Figure 3). Evidence is available that the thiol/disulfide state of vWF multimers may serve as a critical regulator of vWF activity, which is supported by many experimental observations [45,62,63]. In the case of plasma vWF binding to ULvWF strings attached to an endothelial surface, vWF self-association was mediated by covalent interaction, as the formation and propagation of ULvWF strings were dose-dependently reduced by blocking thiols on vWF with N-ethylmaleimide (NEM) [64]. Further studies unveiled that the mechanism underlying formation of the vWF strings involves in cysteine thiol/disulfide exchange as the formation of the strings was inhibited by thiol alkylation [61] and the string structure was disrupted by the small thiol, N-acetylcysteine (NAC) [62]. Xie *et al*. hypothesized that redox regulation was possibly involved in further controlling of vWF multimer size [65]. This group claimed that a protein disulfide bond reductase in conditional medium from in vitro cultures of various endothelial cell lines could reduce plasma vWF multimer size associated with formation of new thiols and that its action could be ablated by the thiolblocking agents, iodoacetamide, NEM or E-64. As mentioned above, this active component has been isolated from human endothelial cell conditional medium and shown to be the trimeric glycoprotein, TSP-1. TSP-1 reduces the average multimer size of vWF secreted by endothelial cells through forming thiol-dependent complexes of TSP-1 and vWF. Moreover, vWF-reducing activity of TSP-1 is in the calcium-binding/C-terminal sequence and required a free thiol at position 974 [66]. It was confirmed that the higher the plasma TSP-1/vWF molar ratio, the smaller the average vWF multimer size [67]. Surprisingly, two TSP-1 knock-out mice studies showed that TSP-1 might actually keep unfolded endothelium-bound and subendothelial VWF from degradation by plasma ADAMTS13, possibly by competing for A3 domain within vWF [68,69]. However, it is important to note that both TSP-1 and vWF stored in platelet α-granules are released upon platelet activation [8,70]. TSP-1 influences plasma and platelet vWF multimeric size differently and may be more relevant for the control of the vWF release from platelets (Figure 3). Meanwhile, there were the parallel and cooperative evidences that the disulfide-bond-reducing activity of ADAMTS13 may prevent covalent lateral association and increase platelet adherence of plasma-type vWF multimers induced by high fluid shear stress [47]. ADAMTS13 contains TSP1 repeats and cysteine-rich domain [71-73]. The cysteine-rich, TSP1 repeats and CUB domain located at C-terminal region of ADAMTS13 primarily target disulfide bonds in the C-domain of plasma vWF multimers induced by high shear stress, thus impeding covalent lateral association and decreasing platelet recruitment on plasma vWF multimers [74]. Recent insights in the vWF self-association led to the identification of cysteine thiols targeted by ADAMTS13 that resides in the vWF C-domain [18]. Alkylation assay by MPB revealed that free thiols buried in the native protein were detected in vWF in the presence of SDS and in the condition of heat [75]. 

Intriguingly, mature vWF, both purified from plasma and made recombinantly, contains unpaired cysteine thiols [61,74,75] that are localized in the N terminal of D3 domain and C-terminal of C domains [57,61,74]. The entire array of cysteines in vWF is listed in Table 1. Nine free Cysteine thiols in plasma vWF are in the D3 and C domains, with seven of the nine centering on the C domain [61]. In contrast to plasma vWF, ULvWF did not contain surface exposed thiols [61]. And the adhesion of plasma vWF to ULvWF secreted from histamine stimulated HUVECs could be ablated by the thiol blocking agents NEM and MPB, which could also directly block the formation of ULvWF strings [57]. The shear focre on vWF generated by a cone and plate viscometer could promote the formation of disulfide bonds by decreasing thiol exposure. And likewise, the shear-induced binding of vWF to platelets was blocked by MPB, which predominantly binds to the exposed thiols [61]. Recently, a thiol/disulfide mechanism of vWF self-association was elucidated through unpaired cysteines in the C-terminal part of vWF including Cys2431-Cys2453 and nearby Cys2451-Cys2468 by mutagenesis studies [18,61]. In short, Cys2431 thiolate reduced from Cys2431-Cys2453 disulfide bond by a reductase in one molecule of vWF attacks the same disulfide bond in another vWF molecule to form the dimmer. Then the Cys2451 thiolate of one of the molecules in the dimer attacks the Cys2431-Cys2453 disulfide bond of the third molecule, adding third vWF molecule to the dimer. Other molecules can then add by the same reaction, thus leading to the formation of vWF multimers [18]. Although Cys2431-Cys2453 in the C2 domain contributes to self-association of vWF, the mature monomeric vWF subunit contains 169 cysteines that are involved in either intra-subunit or inter-subunit vWF disulfide bond formation [36], as shown in Table 1, and there might be other cysteines in vWF involved in self-association. 

**Table 1:** Potential cysteines involved in vWF self-association

**Domain region Identified disulfide bond/cysteines in vWF multimers**				
D’ domain	767 ↔ 808	776 ↔ 804	810 ↔ 821	
D3 domain	867 ↔ 996	889 ↔ 1031	898 ↔ 993	914 ↔ 921
	1060 ↔ 1084	1071 ↔ 1111	1089 ↔ 1091	1126 ↔ 1130
	1149 ↔ 1169	1153 ↔ 1165	1196 ↔ 1199	1234 ↔ 1237
	**889, 898**			
A domain	1272 ↔ 1458 (A1)	1669 ↔ 1670 (A2)	1686 ↔ 1872 (A3)	
A3-D4 domain	1879 ↔ 1904	1899 ↔ 1940	1927 ↔ 2088	
D4 domain	1950 ↔ 2085	1972 ↔ 2123	1993 ↔ 2001	
C domain	2724 ↔ 2774	2739 ↔ 2788	2750 ↔ 2804	2754 ↔ 2806
	**2448, 2451**	**2490, 2491**	**2453, 2528**	**2533**

“↔” represents disulfide bond, free cysteines are shown in bold. The sequence locations include the signal peptide and propeptide sequences.

### The synergistic mechanism of the main platelet receptors involved in vWF self-association

**Figure 4:**
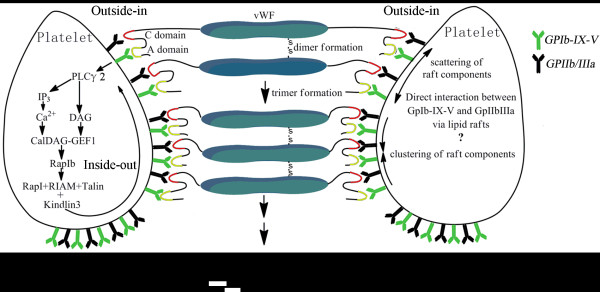
**Proposed mechanism of associated vWF activating GPIIb/IIIα.** Lateral associated vWF are formed by different multi-vWF through disulfide bond. Associated vWF binds to GPIb-IX-V through A1 domain (in yellow), leading to a shift of GPIIb/IIIa conformation from a low-affinity to high affinity. After the conformation alteration, activated GPIIb/IIIa binds to the C domain (in red) of associated vWF by RGDS within C domain. The generally known signaling pathway from GPIb-IX-V to GPIIb/IIIa is shown in the left platelet which depends on the out-inside and inside-out signaling. Here we have depicted the events subsequent to the interaction between vWF and GPIb-IX-V. The activated GPIb-IX-V subsequently increases the level of intracellular calcium, which is regulated by PLCγ2 derived second messengers inositol 1,4,5 trisphosphate (IP3) and diacylglycerol (DAG). The elevated Ca2+ and DAG together increase CalDAG-GEFI activity that in turn activates RapIb. This leads to the formation of an “activation complex” consisting of RapI, RIAM and talin, which finally cooperates with kindlin3 to activate GPIIb/IIIa. Another possible pathway from GPIb-IX-V to GPIIb/IIIa is shown in the right platelet. On the other hand, lipid rafts may serve as a platform to concentrate activated GPIb-IX-V and GPIIb/IIIa in their respective local membrane when vWF self-association occurs. The formation of lipid rafts is required for out-inside signaling in contrast with the activation of receptor itself by ligand only. They can change the recruitment of these intracelluar receptors and their adaptor proteins in close proximity manner.

vWF binding to GPIb-IX-V is known to induce activation of GpIIb/IIIa which finally results in GpIIb/IIIa-dependent platelet aggregation [76]. The GPIb-IX-V mediated platelet activation depend on the activation of phospholipase Cγ2 (PLCγ2) after vWF binding [77,78]. Succeedingly, activated PLCγ2 promotes the production of inositol 1, 4, 5 trisphosphate (IP_3_) and diacylglycerol (DAG) by hydrolyzing phosphatidylinositol 4,5 bisphosphate [79]. IP_3_, a small and fast diffusing water soluble molecule, binds to the IP_3_ receptor (IP_3_R) on the platelet dense tubular system (DTS). The IP3R, designated as calciumselective cation channel, allows efflux of Ca^2+^ from the DTS, thus increasing the cytoplasmic Ca^2+^ level [80]. Increased Ca^2+^ level activates “Ca^2+^ and diacylglycerol regulated guanine nucleotide exchange factor I” (CalDAG-GEFI) [81,82], which in turn promotes the activation of Rap1b, a small GTP binding protein. Rap1b is critical for GpIIb/IIIa activation and platelet function [83] by regulating cytoskeletal rearrangements through interactions with Rap1-interacting adaptor molecule (RIAM) [84]. Consequently, activated Rap1 forms an “activation complex” containing Rap 1, RIAM and talin that redistributes to the plasma membrane and activates GpIIb/IIIa [85-87]. Recent evidence has shown that kindlin3 is also involved in GpIIb/IIIa activation, as mice with dysfunctional kindlin3 have defective GpIIb/IIIa activation despite normal talin expression, resulting in severe bleeding and resistance to arterial thrombosis [88]. Therefore, the PLCγ2-dependent mobilisation of calcium from intracellular stores plays a critical role in the activation of GpIIb/IIIa upon vWF binding to GPIb-IX-V (Figure 4) [89]. 

In normal circulation within intact vasculature, most platelets never have the access to interact with the endothelial surface during their whole lifetime. However, at sites of vascular injury, platelets promptly adhere to the subendothelial extracellular matrix bridged by vWF to limit hemorrhage and promote tissue healing [80]. The results were verified by the observation that transient interactions of platelet-receptor GpIb with immobilized vWF mediate the rolling of platelets at sites of vascular damage, during which rolling reduces platelet velocity and prolongs the contact time with reactive components of the cell matrix [90]. Particularly, platelets could readily bind to extremely long “beads-on-a-string” structures of newly released ULvWF formed under laminar flow that were visible by phase-contrast microscopy [13]. It was in line with the study showing that platelets spontaneously bound to a subset of vWF strings secreted from histamine stimulated HUVEC in a GPIb dependent manner [62,91]. Importantly, platelets adhered to ULvWF fibers expressed P-selectin and bound PAC-1 (antibody against GpIIb/IIIa), suggesting that the adhesion of platelets to ULvWF fibers induced rapid platelets activation [48]. Recent elegant studies showed that fluid shear in circulation could increase the effective size of vWF binding to platelet GpIbα via protein self-association, which triggers mechanotransduction and platelet activation by enhancing the drag force applied on the cell-surface receptor, implying that vWF self-association is necessary for platelet activation (Figure 4) [63].

Additionally, lipid rafts may serve as a platform to concentrate activated GpIb-IX-V and GpIIb/IIIa in their respective local membrane when vWF self-association occurs and attaches. The GPIb-IX-V complex was first reported to localize to membrane microdomains by Dorahy *et al*. in 1996 [59], but these researchers did not investigate the functional consequences of this association. Increasing evidence is emerging that GPIb-IX-V and GpIIb/IIIa were reported to localize to lipid rafts by the method of sucrose density centrifugation [92-94]. Although relative quantities of GpIIb/IIIa in raft and non-raft fractions did not change on activated platelet, vWF would increase the percentage of GpIb-IX-V located to lipid raft [93]. Disruption of rafts by cholesterol depletion markedly inhibited virtually every aspect of GPIb-IX-V complex function, which results in the inside-out activation of the integrin GpIIb/IIIa, and subsequent platelet aggregation [93]. We speculated that lipid rafts might play a key role in signaling after engagement of the complex by VWF self-association. Here, a model is proposed to explain effects of lipid rafts formation of on platelet activation upon stimulation by vWF self-association (Figure 4). This model requires that GPIb-IX-V complexes be clustered sufficiently close to allow formation of this lipid rafts-ordered complex, and may explain the requirement of rafts for subsequent GPIIb/IIIa activation. This raft association of GPIb-IX-V induced by self-associated vWF would induce GPIIb/IIIa activation in a way different to the outside-in/inside-out signal pathway in platelet activation discussed above. Therefore, vWF self-association may be a cryptic factor in the signaling pathway of platelet.

## Conclusion

vWF self-association has been become increasingly appreciated over the past several years. The self-association ability of vWF represents an additional mechanism that the circulating multimers interact in a reversible manner with matrix-bound and endogenous subendothelial vWF under conditions of hydrodynamic shear. Conversely, the association is damped by increasing cleavage by ADAMTS13 under fluid shear stress. This reaction is likely to account for a majority of vWF proteolysis after secretion and determines the distribution of circulating vWF multimers in vivo. The other mechanism that has been shown to alter vWF self-association is the reduction of the disulfide-bonds holding the subunits together, which led to the identification of TSP-1 as a disulfide reductase involved in regulation of vWF multimer size. We speculate that ADAMTS13/TSP-1 and vWF self-association work together to regulate vWF multimer assembly and degradation, and functional imbalances between the two sides are related mechanistically to the problems of bleeding and thrombosis. It has been also indicated that a thiol/disulfide mechanism of self-association of vWF involves in either intra-subunit or inter-subunit vWF disulfide bond formation, suggesting the possibility that the cysteines exposed on plasma vWF or buried in might result in self-association. Above all, vWF self-association binding to GPIb-IX-V may trigger outside-in signals of platelet activation through mechanotransduction applied on the cell-surface receptor. In turn, ligand binding to GpIIb/IIIa is controlled by inside-out signals that modulate receptor conformation and clustering. Further understanding of vWF’s dynamic self-association can provide new approaches and theory evidences for a subject with immediate physiological and signaling relevance.

## Abbreviations

vWF: von Willebrand factor; ADAMTS13: A disintegrin and metalloproteinase with a thrombospondin type 1 motif, member 13; TSP-1: Thrombospondin-1; GPIb: Glycoprotein Ib; GPIIb/IIIa: Glycoprotein IIb/IIIa; TTP: Thrombotic thrombocytopenic purpura; vWD: von Willebrand disease; vWFpp: vWF propeptide; WT-vWF: Wild type vWF; ULvWF: Ultra-large vWF; ELISA: Enzyme-linked immunosorbent assay; b-vWF: Biotinylated vWF; HUVECs: Human umbilical vein endothelial cells; NEM: N-ethylmaleimide; NAC: N-acetylcysteine; PLCγ2: Phospholipase Cγ2; IP3: Inositol 1,4,5 trisphosphate; DAG: Diacylglycerol; IP3R: IP3 receptor; DTS: Dense tubular system; CalDAG-GEFI: Ca^2+^ and diacylglycerol regulated guanine nucleotide exchange factorI; RIAM: Rap1-interacting adaptor molecule.

## Competing interests

The authors declare that they have no competing interests.

## Authors’ contributions

All authors participated in manuscript preparation, research design, data collection and analysis, drafting and critical revision of the manuscript. All authors read and approve the final manuscript.
